# 急性髓系白血病（M_3_）伴多发性骨髓瘤1例

**DOI:** 10.3760/cma.j.issn.0253-2727.2023.10.017

**Published:** 2023-10

**Authors:** 彬 闫, 苏慧 刘, 天喜 胡, 林静 陶, 亚平 叶, 媛 周, 伟鹏 杜

**Affiliations:** 1 南阳市中心医院医学检验科，南阳 473000 Department of Clinical Laboratory Medicine, Nanyang Central Hospital, Nanyang 473000, China; 2 南阳市血液病学重点实验室，南阳 473000 Nanyang Key Laboratory of Hematology, Nanyang 473000, China; 3 南阳市中心医院血液内科，南阳 473000 Department of Hematology, Nanyang Central Hospital, Nanyang 473000, China

患者，男，51岁，以间断发热10余天，齿龈出血1 d为主诉于2019年9月3日入院。血常规：WBC 3.20×10^9^/L，RBC 1.43×10^12^/L，HGB 45 g/L，PLT 11×10^9^/L。骨髓象：增生尚活跃。粒系异常增生占59.5％，异常早幼粒细胞占58.0％，POX染色：强阳性。红系增生受抑，仅见6.0％，以中晚红细胞为主，形态大致正常。成熟红细胞部分呈缗钱状排列。淋巴细胞占9.5％，形态大致正常。浆细胞占25.0％，均为原始幼稚浆细胞，可见双核浆细胞。巨核细胞缺如，血小板单个散在少见。骨髓活检：HE及PAS染色示送检骨髓增生极度活跃（>90％），异常细胞弥漫增生，部分细胞胞体大，胞质量中等至丰富，胞核不规则，核染色质细致；部分细胞胞体大，胞质丰富，核偏位，部分可见核仁，少量偏成熟阶段粒红细胞散在分布，巨核细胞偏少，分叶核为主；纤维组织增生。网状纤维染色MF-2级。免疫分型：髓系约占66.0％，主要表达CD9、CD13、CD15、CD33、CD38、CD58、CD64、CD117、CD123、MPO。CD45阴性且SSC较淋巴细胞稍大的分布区域可见异常细胞群，表达CD38、CD56、CD117、cKappa，考虑为异常浆细胞。多重巢式RT-PCR检测PML-RARα（bcr1）融合基因阳性，余AML相关基因阴性，荧光原位杂交（FISH）发现t（15;17），AML常见基因突变筛查低水平FLT3/ITD突变，染色体核型筛查结果示46，XY[6]。符合APL［PML-RARα（Bcr1），中危，FLT3-ITD（+）］。

患者血清白蛋白41.7 g/L，β-微球蛋白3.36 µg/ml，LDH 343.0 U/L，IgG 5.37 g/L，IgA 20.2 g/L，IgM 0.28 g/L，游离轻链κ 10 980 mg/L，λ 6.12 mg/L。血清蛋白电泳可见异常单克隆免疫球蛋白条带，M蛋白定量11.0 g/L。血尿免疫固定电泳可见伴κ型M蛋白阳性。浆细胞FISH结果显示del（17p）、t（4;14）、1q21、1p32以及IgH-FGFR3/t（4;14）（p16;q32）。影像学：MRI所见诸椎体及附件弥漫性信号异常，符合血液系统疾病表现；T8、L2椎体上形态及信号异常，压缩性骨折可能性大；脊柱退行性变。头颅正侧位及骨盆诸骨X-线未见明显骨质异常。因此，患者符合MM IgA κ型（DS：Ⅲ期；ISS：Ⅰ期；R-ISS：Ⅱ期；mSMART危险分层：高危；三打击）。

患者入院确诊后首先给予全反式维甲酸联合亚砷酸28 d。结束后患者血常规基本恢复正常，APL骨髓象获得完全缓解（CR），PML-RARα融合基因定量降为8.14％，在第1次巩固治疗结束后PML-RARα融合基因定量为阴性，流式细胞术检测未见异常早幼粒细胞，现标准的巩固和维持治疗已结束，持续观察中。患者MM的治疗始于APL双诱导结束后，骨髓象浆细胞升至67.0％，多为原始、幼稚浆细胞，血清M蛋白量已升至13.0 g/L，游离轻链κ也升至18 300 mg/L，排除反应性浆细胞增高，同时提示患者的MM病情进展。首先给予VD方案（硼替佐米+地塞米松），待第1个疗程结束后继续VRD-lite方案（硼替佐米+来那度胺+地塞米松），在第4疗程结束后患者MM已达严格意义的CR，此时流式细胞术残留浆细胞（MRD）<0.01％。在继续1个疗程的VRD方案后行自体造血干细胞移植，输注CD34^+^HSC共2.00×10^6^/kg。白细胞和血小板于输注后11 d植活，并顺利出仓。随后由于经济原因，患者拒绝二次移植。在继续6个疗程VRD方案后，患者的MM有复发征象，流式细胞术MRD检测单克隆浆细胞0.27％，继续1个疗程VRD方案后升至5.11％，改用PVD方案（硼替佐米+泊马度胺+地塞米松）联合达雷妥尤单抗后降为1.27％，此外血清和尿蛋白电泳转阴，但血清和尿免疫固定电泳仍可见κ型单克隆免疫球蛋白条带。后续患者拒绝了达雷妥尤单抗的继续应用，目前采用PVD方案治疗中。

